# Comparison between cultured and wild Pacific white shrimp (*Penaeus vannamei*) vitellogenesis: next-generation sequencing and relative expression of genes directly and indirectly related to reproduction

**DOI:** 10.7717/peerj.10694

**Published:** 2021-02-23

**Authors:** Araceli Lorena Montes-Dominguez, Jesus Arian Avena-Soto, Jorge Luis Lizarraga-Rodriguez, Rodrigo de Jesus Perez-Gala, Stephanie Jimenez-Gutierrez, Jesus Alberto Sotelo-Falomir, Fernando Marino Pinzon-Miranda, Francisco Martinez-Perez, Horacio Alberto Muñoz-Rubi, Dario Chavez-Herrera, Laura Rebeca Jimenez-Gutierrez

**Affiliations:** 1 Facultad de Ciencias del Mar, Universidad Autonoma de Sinaloa, Mazatlan, Sinaloa, Mexico; 2 Fitmar, Proveedora de larvas S.A. de C.V., La Guasima, Sinaloa, Mexico; 3 Laboratorio de Genomica de Celomados, Universidad Industrial de Santander, Bucaramanga, Santander, Colombia; 4 Centro Regional para la Investigacion en Acuicultura y Pesca, Instituto Nacional de Pesca y Acuacultura, Mazatlán, Sinaloa, México; 5 CONACyT, Direccion de Catedras-CONACYT, CDMX, Mexico

**Keywords:** Circadian rhythm, Transcriptome, Cultured shrimp, Relative expression, Vitellogenesis, Wild shrimp

## Abstract

Shrimp fisheries are among the most important fisheries worldwide, and shrimp culture has increased considerably in recent years. Most current studies on reproduction-related genes have been conducted on cultured shrimp. However, gene expression is intimately linked to physiological and environmental conditions, and therefore an organism’s growth environment has a great influence on reproduction. Thus, gene expression profiling, should be applied in fisheries studies. Here, we identified the expression patterns of 76 reproduction-related genes in *P. vannamei* via the analysis of pooled transcriptomes from a time-series experiment encompassing a full circadian cycle. The expression patterns of genes associated both directly (*Vtg*, *ODP*, and *ProR*) and indirectly (*FAMet*, *CruA1*, and *CruC1*) with reproduction were evaluated, as these genes could be used as molecular markers of previtellogenic and vitellogenic maturation stages. The evaluated genes were prominently upregulated during vitellogenic stages, with specific expression patterns depending on the organism’s environment, diet, and season. *Vtg*, *ProR*, *ODP*, and *FaMet* could serve as molecular markers for both wild and cultured organisms.

## Introduction

Industrial shrimp fisheries are among the most important fisheries worldwide (FAO, 2020; Tirado-Ibarra et al., 2020). However, most biological processes have been studied in cultured organisms, due to the growing importance of the shrimp culture industry (CONAPESCA, 2020). As a result, reproduction-associated genes have been well characterized in cultured white shrimp *Penaeus vannamei* (Uengwetwanit et al., 2018; Wang et al., 2019) but not in wild organisms. Therefore, standardizing molecular approaches for gene expression analysis in wild organisms is crucial.

Reproduction studies in wild shrimp are based on morpho-colorimetric and histological primary analyses of the maturity stages, which characterize changes in gonadal and the oocyte development, respectively. These evaluated responses depend on the molecular mechanisms of vitellogenesis, which modulate the process of yolk synthesis and its accumulation in the oocyte and therefore also govern growth (Chen et al., 2018).

Molecular studies on crustacean reproduction primarily focus on gene characterization, as well as mRNA expression and transcriptome analyses of different vitellogenesis stages; which involves vitelline protein production via endoproteolysis of vitellogenin (Vtg; i.e., the precursor of vitelline protein). Therefore, Vtg is the most studied reproduction-related gene due to its role as the most important nutrient source for embryo development (Thongda et al., 2015; Boulangé-Lecomte et al., 2017; Jimenez-Gutierrez et al., 2019).

However, other ovary expressed genes are known to participate in reproduction regulation in crustaceans either directly or indirectly, including genes associated with gonadal maturation, physiological processes, among others, in addition to some genes in the hepatopancreas that regulate extraovarian Vtg sources and other nutrients (Shen et al., 2014; Jimenez-Gutierrez et al., 2019). Additionally, the X organ/sinus gland complex located in the eyestalk also regulates vitellogenesis and molting through hormone secretion (Bai et al., 2015). Previously reported transcriptomes feature between 25 and 33 reproduction-related genes (Gao et al., 2014; Jimenez-Gutierrez et al., 2019), some of which have been used as molecular markers of ovarian maturation, particularly *Vtg*, *Vtg receptor* (*VtgR*), *gonadotropin-releasing hormone receptor* (*GnRHR*), *Vigillin*, and *Torso-like*, among others (Shen et al., 2014; Tarrant et al., 2014; Uengwetwanit et al., 2018).

Additionally, many other *P. vannamei* genes have been previously characterized via next-generation sequencing; however, several of these genes have never been used as molecular markers (Jimenez-Gutierrez et al., 2019). In addition to *Vtg*, the most important genes directly involve in reproduction regulation include the *ovary developing protein* (*ODP*; which modulates oocyte maturation) and *progesterone receptor* (*ProR*). Moreover, other protein encoding genes indirectly involved in reproduction by regulating nutrient sources (this parameter constitutes the main difference between aquaculture and wild environments), are *farnesoic acid O-methyltransferase* (*FAMet*; which could have a role in reproduction and growth), and *crustacyanins A1* and *C1* (*CruA1* and *CruC1*, respectively), which bind astaxanthin carotenoids (Gamiz-Hernandez et al., 2015; Meléndez, 2017). Therefore, our study sought to identify novel genes involved in reproduction regulation through next-generation sequencing, as well as their contribution to *P. vannamei* reproduction control and the expression of new molecular markers in cultured and wild organisms.

## Materials and Methods

### Experimental organisms

All animals were handled according to ARRIVE (Animal Research: Reporting of In Vivo Experiments) guidelines. Organisms were collected from two sources. Cultured *P. vannamei* specimens were obtained in November, 2018 from Fitmar farm (El Walamo, Mazatlan, Mexico). The organisms were kept under controlled conditions at 28 °C, a pH of 7.6 and 35 PSU (practical salinity units). Wild organisms, on the other hand, were donated by the Regional Center for Aquaculture and Fisheries Research of Mazatlan (Instituto Nacional de Pesca y Acuacultura; field study approval number PPF/DGOPA-002/18). These organisms were collected from the East Pacific (23°20′N 106°30′W) during open season (September, 2018 to March, 2019; INAPESCA, 2019). Two samplings were performed on November, 2018 (25.76 ± 0.89 °C and 35  ± 0.1 PSU) and March, 2019 (20.32 ± 1.33 °C and 34.9 ± 0.2 PSU). On both occasions, the shrimps were identified to the species level (Perez-Farfante & Kensley, 1997), and immediately frozen while still in the vessel.

Only mature females (56.48 ± 9.6 g and 18.92 ± 1.68 cm total length) from both organism sources were examined. The females were euthanized on ice and immediately measured and dissected thereafter. The ovary stages were primarily identified via morpho-colorimetric methods (Pérez-Ferro & Paramo-Granados, 2014). Hepatopancreas and eyestalk samples dissected from cultured organisms were kept at −80 °C until required for molecular analyses, whereas the ovaries were extracted and preserved separately for other procedures. A fragment of the ovarian tissue was preserved in Davidson’s solution (Bell & Lightner, 1988), for ovarian stage confirmation via histological methods (Bell & Lightner, 1988; Alfaro-Montoya, 2013) with hematoxylin and eosin staining (Humason, 1979). The rest of the ovary tissues were kept at −80 °C until used for molecular analyses. Complete ovaries dissected from wild organisms, were kept at −80 °C until required.

### RNA isolation and illumina sequencing

To obtain mRNA transcription profiles throughout an entire circadian cycle under controlled conditions, only cultured organisms were used. For this purpose, eight samplings were conducted every three hours and five cultured females were collected per sampling for a total of 40 organisms. Total RNA from each tissue (ovaries, hepatopancreas and eyestalk) was obtained according to Jimenez-Gutierrez et al. (2019), and a pool from the 40 organisms for each tissue was submitted to Genoma Mayor, Universidad Mayor in Chile (Santiago de Chile) for next-generation sequencing. The library was constructed via the TruSeq Stranded mRNA (Illumina, San Diego, CA) protocol and sequenced in an Illumina MiSeq instrument according to the manufacturer’s instructions. *De novo* assembly and bioinformatic analyses were also conducted as previously described (Jimenez-Gutierrez et al., 2019).

### mRNA expression of *P. vannamei* reproduction regulation genes

Based on our histologic results, the samples from wild and cultured organisms were classified as previtellogenic and vitellogenic ovaries. Stage III and post-spawning stages were omitted, as they were considered intermediate stages. A 3 × 2 factorial experimental design was used; with three different collection conditions and two vitellogenic stages. Moreover, given that cultured shrimp and *Vtg* represent the most well characterized organism source and reproduction-associated gene, respectively, these two were used as sample source and molecular marker controls.

Total RNA was extracted from at least four organisms from each evaluated condition in duplicate, and cDNA was synthesized with the RevertAid First Strand cDNA Synthesis Kit (Thermo scientific), according to the manufacturer’s instructions. *Vtg*, *ODP*, and *ProR* were deemed direct reproduction modulators, whereas *FAMet*, *CruA1*, and *CruC1* were considered to indirectly regulate *P. vannamei* reproduction. Both *L8* and *β-actin* were used as housekeeping genes for relative gene expression analysis.

cDNAs were amplified using the TopTaq Master Mix Kit (QIAGEN) according to the manufacturer’s instructions using the specific Tm for each gene (Supplemental Information 1). For gen expression analyses, 600 ng of total RNA template were employed for all target genes, whereas, 300 and 400 ng were used for *L8* and *β-actin*, respectively. PCR products for each gene were first purified with the QIAquick PCR Purification Kit (Nucleospin), according to the manufacturer’s instructions and quantified with a spectrophotometer. All PCR products were electrophoretically separated (including the purified gene products), digitalized in the Gel Doc EZ System (Biorad), and analyzed with the ImageLab software.

Absolute expressions were quantified based on previously purified PCR products. The relative expression of each target gene was calculated via the 2^−ΔΔ*Ct*^ method using the housekeeping genes and previtellogenic stage as first and second delta, respectively (Schmittgen & Livak, 2008), with modifications. Unlike Ct values, where lower values indicate higher mRNA concentrations and vice versa, absolute expression provides a direct gene expression quantification value. The following modification allows for the use of the absolute expression in the 2^−^ΔΔCtm^^ formula:

\begin{eqnarray*}& & Ctm=30-\mathrm{AE} \end{eqnarray*}

where Ctm is the modified Ct; 30 is a constant that represents the number of PCR cycles; AE is the absolute expression of each PCR product.

### Statistical analysis

Relative expressions for each condition were analyzed for normality and variance homogeneity. Statistical significance was evaluated via one-way ANOVA (*P* ≤ 0.05) and differences between treatments were determined via Tukey-Kramer multiple-comparison tests. Finally, correlation coefficients from multivariate Principal Component Analysis (PCA) were used to verify the relationships between the evaluated genes using the InfoStat 2018 statistical software (Di Rienzo et al., 2018).

## Results

### Histological results from ovary development

Upon analyzing all samples to characterize ovary development, five development stages and one post-spawning stage were identified. In previtellogenic stages I and II, germinal tissue, follicular cells, perinuclear oocytes, and cortical alveolus were observed. Stage III is a transition between previtellogenic and vitellogenic oocytes, and lipid droplets and perinuclear alveoli were observed in the first vitellogenic oocytes.

**Figure 1:**
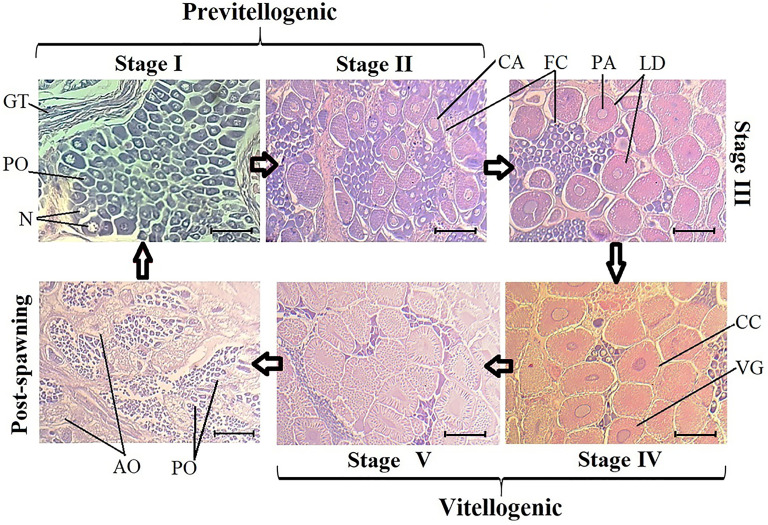
Histological sections of oocyte differentiation in ovaries from *Penaeus vannamei*. GT, germinal tissue. PO, perinuclear oocytes. N, Nucleus. CA, cortical alveolus. FC, Follicular cells. PA, perinuclear alveolus. LD, lipid drops. CC, cortical crypts. VG, vitellic grains. AO, atresic oocytes. Scaled bar represent 20 µm.

Vitellogenic stages IV and V exhibited larger oocytes, with elongated nuclei, vitellogenic grains, and cortical crypts. Finally, in the post-spawning stage, flaccid tissue, atretic oocytes, and a group of stage I oocytes began to appear (Fig. 1). The V-Red semi-stage, where stage V ovaries turn red, was only observed in wild organisms from samples acquired in March (Supplemental Information 2).

**Figure 2:**
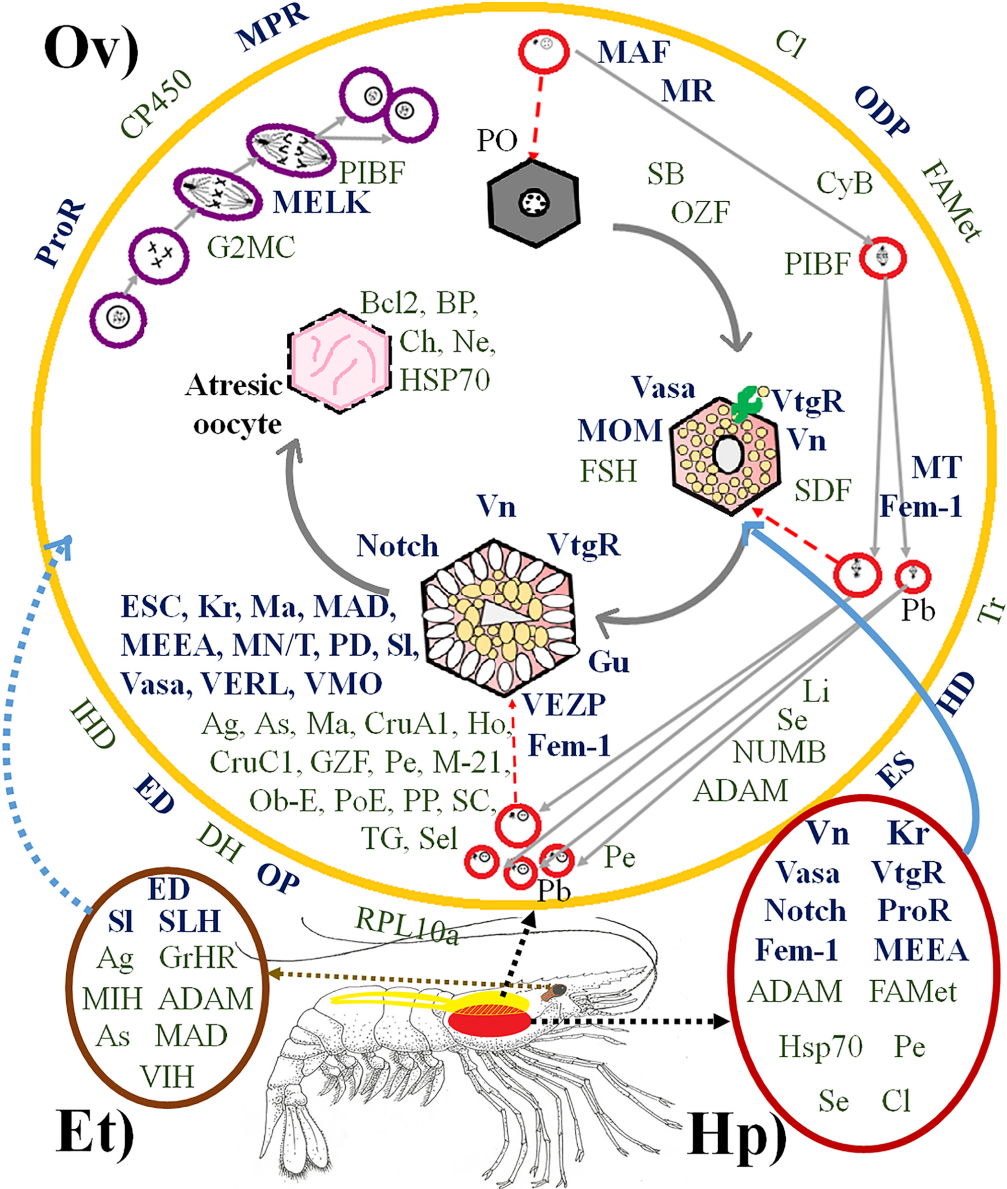
Proteins directly and indirectly related to *Penaeus vannamei* reproduction regulation. (Ov) Ovary, represented in yellow. (Hp) Hepatopancreas, represented in dark red. (Et) Eyestalk, represented in brown. Violet cells represent mitotic process. Red cells represent meiotic process. Hexagonal cells represent oocyte development. Proteins in bold dark blue are directly reproduction-related. Proteins in green are indirectly reproduction-related. Proteins outside the ovary are involved in several places of the ovary at the same time. PO, Primary oocyte. Pb, Polar bodies. Abreviated Genes: Ag, Agrin. As, Asterix. BP, Blastula protease. Ch, Chitinase. Cl, Clathrin. CruA1, Crustacyanin A1. CruC1, Crustacyanin C1. CP450, Cytochrome P450. CyB, Cyclin B. DH, Diuretic Hormone. ED, Estradiol 7 beta Dehydrogenase. ES, Estrogen Sulfotransferase. ESC, Extra sex comb. FAMet, Farnesoic Acid O-Methyltransferase. Fem-1, Sex-determining protein Fem-1. FSH, Female Sterile Homeotic. G2MC, G2 mitotic specific cyclin. GrHR, Gonadotropin-releasing hormone receptor. Gu, Gustavus. GZF, Gastrula zinc finger. HD, Hydroxysteroid Dehydrogenase. Ho, Homeobox. Hsp70, Heat Shock Protein 70. IHD, Inactive Hydroxyesteroid Dehydrogenase. JH, Juvenile hormone epoxide hydrolase. Kr, Krueppel. Li, Lin-9. MN/T, Complex Mago Nashi/TsunagiY14. M-21, Mab-21. Ma, Masquerade. MAD, Mothers Against Decapentaplegic. MAF, Meiosis arrest female. MEEA, Maternal effect embryo arrest. MELK, Maternal Embryonic Leucine zipper Kinase. MIH, Molt-inhibiting hormone. MOM, Missing oocyte meiosis. MPR, Membrane progestin receptor. MR, Meiosis regulator. MT, Maternal protein Tudor. Ne, Neuroparsin. Notch, Notch proteins (Notch, Notchless protein and Strawberry Notch). Ob-E, Obstructor-E. ODP, Ovary Development Protein. OP, Ovaric Peritrophin. OZF, Oocyte zinc finger. PD, Partitioning Defective. Pe, Pelota protein. PIBF, Progesterone-Induced Blocking Factor. PoE, Purity of essence. PP, Peter Pan. ProR, Progesterone Receptor. RPL10a, Ribosomal Protein 10a. SB, Singles Bar. SC, Shuttle craft. SDF, Se: Sel-1. Sel, protein Seele. Sex Determination Fruitless. Sl, Slowmo. SLH, Sex-lethal homolog. TG, Twisted Gastrulation. Tr, Trithorax. Vasa, Vasa protein. VERL, Vitelline envelope receptor of lysin. VIH, Vitellogenesis-inhibitinghormone. VMO, Vitelline Membrane Outer layer. Vn, Vitellin. VtgR, Vitellogenin Receptor. (Artist: Rafael Serrano-Quiñonez).

### *P. vannamei* ovary, hepatopancreas, and eyestalk transcriptomes

The transcriptomes from the ovary, hepatopancreas and eyestalk circadian rhythm pools exhibited 76 genes related to *P. vannamei* reproduction regulation, 32 and 44 of which were directly and indirectly related to reproduction, respectively. As expected, most of them were only found in the ovary transcriptome. Additionally, 14 of them were expressed in both the ovary and hepatopancreas, of which six were expressed in both the ovary and eyestalk, and five were expressed exclusively in the eyestalk (Fig. 2). Among all identified genes, some had never been reported in the GenBank database. The codified proteins were found to be related to one or more steps of the mitotic or meiotic processes, and some of them were specifically involved in oocyte development, such as Vitelline (for which the precursor is Vtg), VtgR, Vasa, Missing oocyte meiosis, TsunagiY14, Fem-1, Crustacyanin A1 and C1, among others.

Some other encoded proteins were simultaneously involved in several parts of the ovary, such as ODP, FAMet, ProR, Membrane progestin receptor, diuretic hormone, ovarian peritrophin, trithorax, among others. The function of each gene is summarized in Supplemental Information 3.

### Relative expression of *Vtg*, *ODP*, *ProR*, *FAMet*, *CruA1*, and *CruC1*

**Figure 3:**
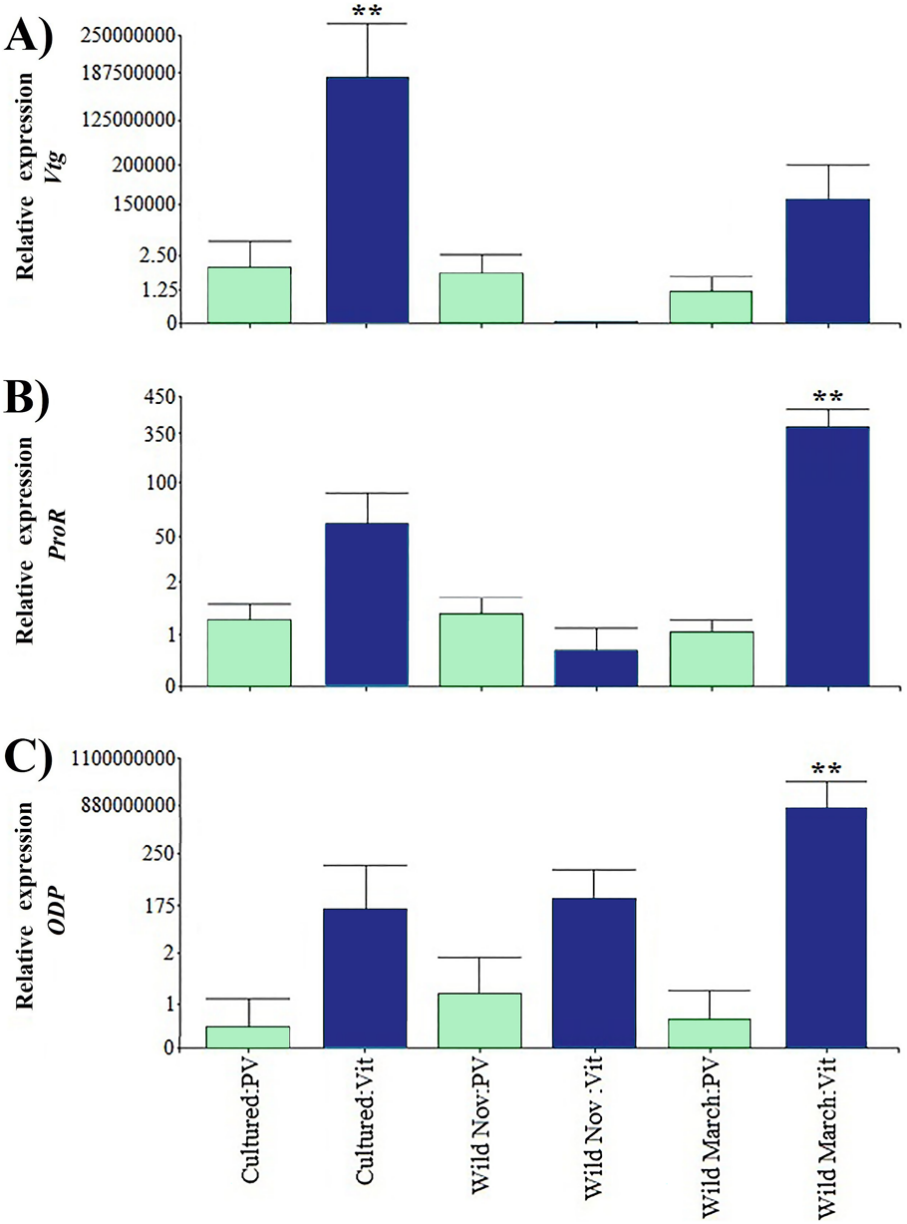
Relative expression of directly shrimp reproduction-related genes. Bars indicates average ± standard error. *n* = 4. Light green bars indicate previtellogenic states. Dark blue bars indicate vitellogenic states. Asterisks (**) indicate highly statistically significant differences at *P* ≤ 0.01. (A) *Vtg*. (B) *ProR*. (C) *ODP*.

Relative expression of directly related reproduction regulation genes was greater than that of indirectly related genes; *Vtg* displayed the highest expression levels among all the evaluated genes (Fig. 3). As expected, *Vtg* expression was greater in vitellogenic stages than in previtellogenic stages; this was more evident in cultured organisms with highly significant differences (i.e., three orders of magnitude greater than the vitellogenic stages of wild-caught shrimp captured in March and eight orders of magnitude greater than the rest of the treatments).

A different pattern was observed in *ODP* and *ProR*, where the highest expression was seen in wild shrimp caught in March during vitellogenic stages, exhibiting two and six orders of magnitude upregulation relative to the rest of the treatments, respectively. However, gene expression was generally lower in previtellogenic stages.

**Figure 4:**
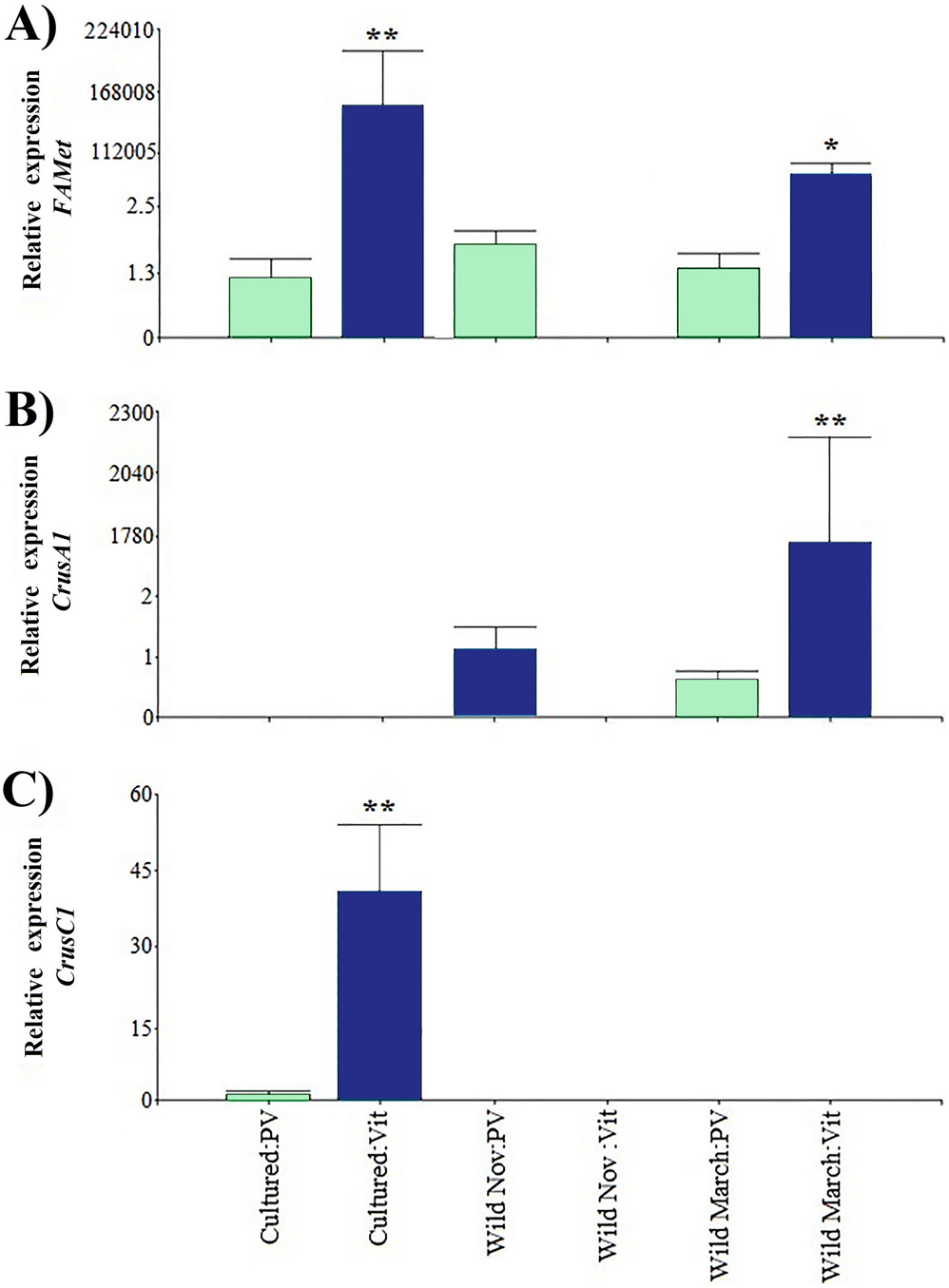
Relative expression of indirectly shrimp reproduction-related genes. Bars indicates average ± standard error. *n* = 4. Light green bars indicate previtellogenic states. Dark blue bars indicate vitellogenic states. Two asterisks (**) indicate highly statistically significant differences at *P* ≤ 0.01. One asterisk (*) indicates statistically significant differences at *P* ≤ 0.05. (A) *FAMet*. (B) *CrusA1*. (C) *CrusC1*.

Regarding the genes that were indirectly associated with reproduction, the relative expression of *CrusC1* was the lowest. *FAMet* showed the same pattern as *Vtg* expression in cultured vitellogenic stages; however, it did not present significant differences in the vitellogenic stages of wild shrimp caught in March, and both treatments had expression values five orders of magnitude higher than those of previtellogenic stages (Fig. 4).

Furthermore, both crustacyanin genes exhibited a particular expression pattern. Specifically, *CrusA1* was only found in wild organisms, whereas *CrusC1* was only found in cultured organisms. As in the previously discussed genes, the previtellogenic stages exhibited highly significant minimum expression values, with three and one orders of magnitude of difference, respectively.

**Figure 5:**
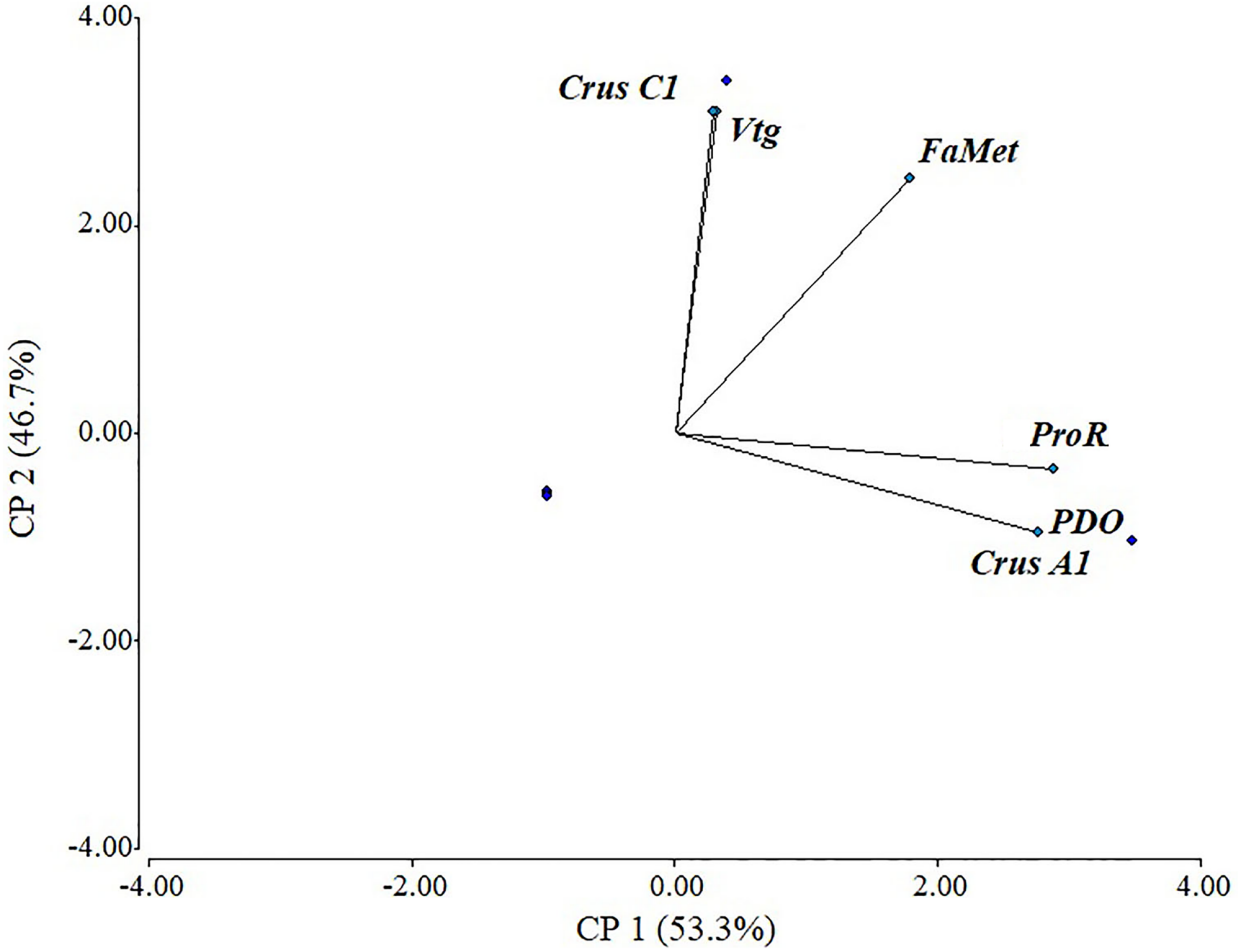
Scaled principal component of relative expression of directly and indirectly shrimp reproduction-related genes.

There was a positive correlation in most evaluated genes, with a cophenetic correlation of 1 (Fig. 5). Correlations between variables were particularly strong between *Vtg* and *CrusC1* (1.0), followed by *ProR*, *CrusA1*, and *ODP* (0.98), and finally *FAMet* and *Vtg* (0.85). Although some variables exhibited a negative correlation (e.g., *CrusC1* and *CrusA1*), none were statistically significant.

## Discussion

Depending on the authors, ovarian development in shrimp is often classified into three to five stages (Perdichizzi et al., 2012; Pérez-Ferro & Paramo-Granados, 2014; Nguyen et al., 2018) plus one post-spawning stage. However, wild shrimp presented a V-Red semi-stage in organisms sampled in March, which was likely due to the rich carotenoid diet of shrimp in spring (Yanar, çelik & Yanar, 2004). Red ovaries had not been reported in cultured organisms. Here, reproduction-related differences between cultured and wild organisms were observed at the anatomical and molecular level.

Transcriptome analysis throughout an entire circadian cycle in cultured organisms allowed for the discovery of a greater number of reproduction-related genes than in previous reports (Gao et al., 2014; Jimenez-Gutierrez et al., 2019). However, infradian rhythms and different collection areas must also be considered to obtain a better estimation of the number of these genes, without considering the large number of genes whose functions remain unknown.

Moreover, a close anatomical and molecular relationship were observed between the ovaries and hepatopancreas, as evidenced by the great number of genes involved in *P. vannamei* reproduction expressed in the hepatopancreas, as well as the presence of *ProR* and *VtgR* in both tissues, thereby demonstrating communication between membranes. The genes examined in this work were divided into directly related reproduction genes, whose encoded proteins have a direct function in oocyte maturation or meiosis, and indirectly related genes, whose encoded proteins bind to directly related proteins, thereby inhibiting or activating them.

Most differential expression reports are based on directly related genes, which are used as molecular markers (Bae et al., 2017; Boulangé-Lecomte et al., 2017; Chen et al., 2017). The differences in the relative expression of the genes in both groups were of several orders of magnitude, with *Vtg* being the most highly expressed, as expected.

*Vtg* expression is known to be species- and tissue-specific, in addition to the effects of external and physiological conditions (Jimenez-Gutierrez et al., 2019). This was confirmed by the differential expression between wild and cultured organisms. Furthermore, the remaining evaluated genes in this work had never been examined in the conditions evaluated herein.

Only a few sequences for *ODP* are available in the GenBank database, of which only one corresponds to crustaceans (Shanghai crab *Eriocheir sinensis*; Zhu et al., 2016). Despite its direct relationship with ovary maturation, its expression patterns had never been documented in crustacean species. In this study, *ODP* was confirmed to be linked to both ovary development and seasonal maturity.

Additionally, the seasonal variation of *ProR* in wild organisms was more noticeable than that of *ODP*. *ProR* is not only well known as a membrane surface receptor that is mainly present in follicular cells (Ye et al., 2010; Wu et al., 2014), but also as a transcriptional activator or repressor, involving cofactors or coactivators that regulate many aspects of the female reproductive system (Klotzbücher et al., 1997; Chen et al., 2008). Its role as a reproduction regulator in the estrogen pathway in vertebrates and invertebrates is well documented in the ovary, as well as its synthesis in the hepatopancreas (Ye et al., 2010; Wu et al., 2014; Thongbuakaew et al., 2016).

This receptor belongs to the steroidogenic-related proteins including estradiol 17 beta dehydrogenase, estrogen sulfotransferase, and hydroxysteroid dehydrogenase, all of which directly modulate estrogen enzyme biosynthesis, and cytochrome P450, which modulates these mechanisms indirectly (James & Boyle, 1998; Thongbuakaew et al., 2016; Subramoniam, 2017). All genes listed above were also found in this study. The relationship between Vtg during the ovarian cycle with progesterone levels and the aforementioned enzymes have been previously reported (Subramoniam, 2017).

Progesterone translocates from follicular cells through the oocyte plasma membrane and binds to ProR to form a progesterone-ProR complex to reach the nuclei and regulate transcriptional activity. Many studies have characterized progesterone activity and several of them have confirmed a considerable increase in progesterone levels during vitellogenesis (Ye et al., 2010; Wu et al., 2014; Thongbuakaew et al., 2016). ProR has been studied via immunohistological approaches (Wu et al., 2014); however, the analysis of its expression had not been implemented for the evaluation of progesterone’s role in ovary maturation or as a molecular marker in crustaceans until now.

Only three indirectly related reproduction genes were evaluated herein due to their key role in nutrition to account for the differences in dietary quality and availability between cultured and wild shrimp. FAMet has been reported to participate in *P. vannamei* reproduction (Hui, Tobe & Chan, 2008). Furthermore, its substrate (i.e., methyl farnesoate) is also related to neurohormones and ecdysteroids (Ye et al., 2010). *FAMet* was evaluated in several tissues, molt conditions, between sexes, and between larvae and juvenile organisms (Hui, Tobe & Chan, 2008); however, its expression in ovaries and its role in gonad maturation had not been characterized. In this work, *FAMet* exhibited the same expression pattern as *Vtg*, and both shared a significant correlation according to our PCA results.

Finally, crustacyanin had not previously been studied as a reproduction-related protein. However, the presence of the V-Red semi-stage warranted the assessment of the aforementioned gene in this work. Crustacyanins are specific crustacean proteins that bind to astaxanthin (Cianci et al., 2002; Budd et al., 2017) and belong to the lipocalin family, which also includes retinol-binding protein (RBP) and apolipoproteins (Ma et al., 2020). There are five precursor subtypes of crustacyanins reported in the GenBank databases: A1, A2, A3, C1, and C2. Nevertheless, only *CruA1* and *CruC1* were found in *P. vannamei* transcriptomes.

These results indicate an evident relationship between the red ovaries from wild organisms caught in March and *CruA1*, suggesting a more intimate link with carotenoids, unlike *CruC1*, which is only found in cultured organisms. The above-mentioned relationships were confirmed by our PCA results. Adult cultured organisms are fed with the same formulated diet throughout most of the year, which is specifically formulated to promote maturation; however, differences between natural diets limit the availability of bioactive metabolites for natural growth and development (Liñán Cabello, Paniagua-Michel & Zenteno-Savín, 2003). Wild shrimp are mostly scavengers that feed on smaller crustaceans, fish, mollusks, plants, and organic detritus (INAPESCA, 2019). Food availability and quality are affected by different population dynamics, regions, and seasons (Yanar, çelik & Yanar, 2004; FAO, 2016).

In this sense, differences in the carotenoid content between cultured and wild shrimp were previously studied, and their possible relationship with gonadal maturation was reported, with extensive focus on wild organisms. This suggests that these carotenoids are able to bind to Vtg to form a lipo-carotene-glyco-protein (Liñán Cabello, Paniagua-Michel & Zenteno-Savín, 2003), which translates into seasonal differences in gonad maturation (Thongda et al., 2015; Jimenez-Gutierrez et al., 2019).

There are several genes involved in shrimp reproduction regulation that possess great potential as molecular markers but are yet to be evaluated and validated. Moreover, despite the wealth of studies that have reported on the physiology and gene expression patterns of cultured shrimp, additional studies should be conducted in wild shrimp to account for the environmental variability that wild organisms face.

## Conclusions

This study identified more than 76 genes related to reproduction regulation in *P. vannamei*. *Vtg* exhibited the highest expression among all evaluated genes. The genes directly- and indirectly-associated with shrimp reproduction studied herein act as molecular markers between previtellogenic and vitellogenic maturation stages. Variations in anatomical and specific gene expression patterns were observed between wild and cultured shrimps. *Vtg*, *ProR*, *ODP*, and *FaMet* could serve as molecular markers for both wild and cultured organisms, whereas *CrusA1* and *CrusC1* were exclusively suitable to characterize wild and cultured organisms, respectively.

##  Supplemental Information

10.7717/peerj.10694/supp-1 Supplemental Information 1 Primers sequences and PCR settings

10.7717/peerj.10694/supp-2 Supplemental Information 2 Colorimetric maturity scale from *Penaeus vannamei* ovaries (A) colorimetric scale. (B) complete gonad from stage V-Red.

10.7717/peerj.10694/supp-3 Supplemental Information 3 Function of reproduction-related proteins Directly related genes.
Indirectly related genes.
Accession numbers of reported genes.

## Additional Information and Declarations

### Field Study Permissions

The following information was supplied relating to field study approvals (i.e., approving body and any reference numbers): Fitmar donated cultured shrimp. The national institute of fishery and aquaculture (Instituto Nacional de Pesca y Acuacultura, Centro Regional para la Investigación en Acuicultura y Pesca) donated wild shrimps from commertial vessels of the country (field study approval number PPF/DGOPA-002/18).

### Data Availability

The following information was supplied regarding data availability: Raw data are available at Figshare: Jimenez-Gutierrez, Rebeca (2020): Relative expression for reproduction genes in Penaeus vannamei. figshare. Dataset. https://doi.org/10.6084/m9.figshare.12420953.v1. The Genbank accession numbers for the analyzed sequences as well as the 76 new sequences are available in Supplemental Files.

## References

[ref-1] Alfaro-Montoya J (2013). Histological description of oogenesis and spermatogenesis in the cultured shrimp, Litopenaeus vannamei. Revista de Biología Marina y Oceanografía.

[ref-2] Bae SH, Okutsu T, Tsutsui N, Kang BJ, Chen HY, Wilder MN (2017). Involvement of second messengers in the signaling pathway of vitellogenesis-inhibiting hormone and their effects on vitellogenin mRNA expression in the whiteleg shrimp, Litopenaeus vannamei. General and Comparative Endocrinology.

[ref-3] Bai H, Qiao H, Li F, Fu H, Sun S, Zhang W, Xiong Y (2015). Molecular characterization and developmental expression of vitellogenin in the oriental river prawn Macrobrachium nipponense and the effects of RNA interference and eyestalk ablation on ovarian maturation. Gene.

[ref-4] Bell TA, Lightner DV (1988). A handbook of normal penaeid shrimp histology.

[ref-5] Boulangé-Lecomte C, Xuereb B, Trémolet G, Duflot A, Giusti N, Olivier S, Legrand E, Forget-Leray J (2017). Controversial use of vitellogenin as a biomarker of endocrine disruption in crustaceans: new adverse pieces of evidence in the copepod *Eurytemora affinis*. Comparative Biochemistry and Physiology. C.

[ref-6] Budd AM, Hinton TM, Tonks M, Cheers S, Wade NM (2017). Rapid expansion of pigmentation genes in penaeid shrimp with absolute preservation of function. Journal of Experimental Biology.

[ref-7] Chen T, Lin T, Li H, Lu T, Li J, Huang W, Sun H, Jiang X, Zhang J, Yan A, Hu C, Luo P. Ren C (2018). Heat Shock Protein 40 (HSP40) in Pacific White Shrimp (*Litopenaeus vannamei*): molecular cloning, tissue distribution and ontogeny, response to temperature, acidity/alkalinity and salinity stresses, and potential role in ovarian development. Frontiers in Physiology.

[ref-8] Chen C, Opazo JC, Erez O, Uddin M, Santolaya-Forgas J, Goodman M, Grossman LI, Romero R, Wildman DE (2008). The human progesterone receptor shows evidence of adaptive evolution associated with its ability to act as a transcription factor. Molecular Phylogenetics and Evolution.

[ref-9] Chen S, Qiao H, Fu H, Sun S, Zhang W, Jin S, Gong Y, Jiang S, Xiong W, Wu Y (2017). Molecular cloning, characterization, and temporal expression of the clock genes *period* and *timeless* in the Oriental river prawn *Macrobrachium nipponense* during female reproductive development. Comparative Biochemistry and Physiology. A: Comparative Physiology.

[ref-10] Cianci M, Rizkallah PJ, Olczak A, Raftery J, Chayen NE, Zagalsky PF, Helliwell JR (2002). The molecular basis of the coloration mechanism in lobster shell: *β*-Crustacyanin at 3.2-Åresolution. Proceedings of the National Academy of Sciences USA.

[ref-11] CONAPESCA (2020). Comisión Nacional de Acuacultura y Pesca. https://www.gob.mx/conapesca/prensa/produce-acuacultura-mexicana-mas-de-400-mil-toneladas-de-pescados-y-mariscos-172466.

[ref-12] Di Rienzo J, Casanoves F, Balzarini MG, Gonzáles L, Tablada M, Robledo W (2018). InfoStat Versión 2018.

[ref-13] FAO (2016). FAO/INFOODS global food composition database for fish and shellfish, version 1.0 -uFiSh1.0.

[ref-14] FAO (2020). Shrimp fisheries under scrutiny. http://www.fao.org/news/story/en/item/10126/icode/.

[ref-15] Gamiz-Hernandez AP, Angelova IN, Send R, Sundholm D, Kaila VR (2015). Protein-induced color shift of carotenoids in *β*-Crustacyanin. Angewandte Chemie International Edition.

[ref-16] Gao J, Wang X, Zou Z, Jia X, Wang Y, Zhang Z (2014). Transcriptome analysis of the differences in gene expression between testis and ovary in green mud crab (*Scylla paramamosain*). BMC Genomics.

[ref-17] Hui JHL, Tobe SS, Chan SM (2008). Characterization of the putative farnesoic acid O-methyltransferase (LvFAMeT) cDNA from white shrimp, Litopenaeus vannamei: Evidence for its role in molting. Peptides.

[ref-18] Humason GL (1979). Animal tissue techiques.

[ref-19] INAPESCA (2019). Análisis de las capturas de camarón en el litoral Pacífico mexicano en la temporada 2018-2019, para implementar el inicio de veda en 2019. Informe Técnico.

[ref-20] James MO, Boyle SM (1998). Cytochromes P450 in crustacea. Comparative Biochemistry and Physiology Part C.

[ref-21] Jimenez-Gutierrez S, Cadena-Caballero CE, Barrios-Hernandez C, Perez-Gonzalez R, Martinez-Perez F, Jimenez-Gutierrez LR (2019). Crustacean vitellogenin: a systematic and experimental analysis of their genes, genomes, mRNAs and proteins; and perspective to next generation sequencing. Crustaceana.

[ref-22] Klotzbücher M, Schwerk C, Holewa B, Klein-Hitpass L (1997). Activation of transcription by progesterone receptor involves derepression of activation functions by a cofactor. Molecular Endocrinology.

[ref-23] Liñán Cabello MA, Paniagua-Michel J, Zenteno-Savín T (2003). Carotenoids and retinal levels in captive and wild shrimp, Litopenaeus vannamei. Aquaculture Nutrition.

[ref-24] Ma H, Sun J, Xu W, Gao W, Hu G, Lai X, Yan B, Gao H (2020). Cloning and functional study of lipocalin: retinol-binding protein-like gene family of the ridgetail white prawn, Exopalaemon carinicauda. Molecular Genetics and Genomics.

[ref-25] Meléndez A (2017). Carotenoides: estructura, propiedades y funciones. En Carotenoides en agroalimentación y salud.

[ref-26] Nguyen TV, Rotllant GE, Cummins SF, Elizur A, Tomer T (2018). Insights into sexual maturation and reproduction in the Norway lobster (*Nephrops norvegicus*) via *in silico* prediction and characterization of neuropeptides and G protein-coupled receptors. Frontiers in Endocrinology.

[ref-27] Perdichizzi A, Pirrera L, Micale V, Muglia U, Rinelli P (2012). A Histological Study of Ovarian Development in the Giant Red Shrimp *Aristaeomorpha foliacea* (Crustacea: Decapoda: Aristeidae) from the Southern Tyrrhenian Sea (Western Mediterranean). Scientific World Journal.

[ref-28] Perez-Farfante I, Kensley B (1997). Penaeoid and sergestoid shrimps and prawns of the world: keys and diagnoses for the families and genera. Mémoires du Muséum National d’Histoire Naturelle, Paris.

[ref-29] Pérez-Ferro DG, Paramo-Granados JE (2014). Maturity Stages of Pink Shrimp *Farfantepenaeus notialis* (Penaeidae) in the Colombian Caribbean. Acta Biologica Colombiana.

[ref-30] Schmittgen TD, Livak KJ (2008). Analyzing real-time PCR data by the comparative C(T) method. Nature Protocols.

[ref-31] Shen H, Hu Y, Ma Y, Zhou X, Xu Z, Shui Y, Li C, Xu P, Sun X (2014). In-depth transcriptome analysis of the red swamp crayfish *Procambarus clarkii*. PLOS ONE.

[ref-32] Subramoniam T (2017). Steroidal control of vitellogenesis in Crustacea: a new understanding for improving shrimp hatchery production. Proceedings of the National Academy of Sciences of the United States of America.

[ref-33] Tarrant AM, Baumgartner MF, Hansen BH, Altin D, Nordtug T, Olsen AJ (2014). Transcriptional profiling of reproductive development, lipid storage and molting throughout the last juvenile stage of the marine copepod *Calanus finmarchicus*. Frontiers in Zoology.

[ref-34] Thongbuakaew T, Siangcham T, Elizur ASuwansa-ArdS, Cummins SF, Sobhon P, Sretarugsa P (2016). Steroids and genes related to steroid biosynthesis in the female giant freshwater prawn, Macrobrachium rosenbergii. Steroids.

[ref-35] Thongda W, Chung JS, Tsutsui N, Zmora N, Katenta A (2015). Seasonal variations in reproductive activity of the blue crab, Callinectes sapidus: vitellogenin expression and levels of vitellogenin in the hemolymph during ovarian development. Comparative Biochemistry and Physiology. A: Comparative Physiology.

[ref-36] Tirado-Ibarra JJ, Jimenez-Gutierrez S, Acuña Carvajal C, Muñoz Garcia I, Martinez-Perez F, Rodriguez-Dominguez G, Perez-Gonzalez R, Jimenez-Gutierrez LR (2020). Crustaceans from shrimp by-catch from the southeastern Gulf of California to the southeastern Mexican Pacific: implications in their community structure and reproduction. Crustaceana.

[ref-37] Uengwetwanit T, Ponza P, Sangsrakru D, Wichadakul D, Ingsriswang S, Leelatanawit R, Klinbunga S, Tangphatsornruang S, Karoonuthaisiri N (2018). Transcriptome-based discovery of pathways and genes related to reproduction of the black tiger shrimp (*Penaeus monodon*). Marine Genomics.

[ref-38] Wang Z, Luan S, Meng X, Cao B, Luo K, Kong J (2019). Comparative transcriptomic characterization of the eyestalk in Pacific white shrimp (*Litopenaeus vannamei*) during ovarian maturation. General and Comparative Endocrinology.

[ref-39] Wu X, Chen H, Liu Z, Cheng Y (2014). Immunorecognition and distribution of progesterone receptors in the Chinese mitten crab *Eriocheir sinensis* during ovarian development. Journal of Shellfish Research.

[ref-40] Yanar Y, çelik M, Yanar M (2004). Seasonal changes in total carotenoid contents of wild marine shrimps (*Penaeus semisulcatus* and *Metapenaeus monoceros*) inhabiting the eastern Mediterranean. Food Chemistry.

[ref-41] Ye H, Song P, Ma J, Huang H, Wang G (2010). Changes in progesterone levels and distribution of progesterone receptor during vitellogenesis in the female mud crab (*Scylla paramamosain*). Marine and Freshwater Behaviour and Physiology.

[ref-42] Zhu L, She T, Zhang Y, Li S, Xu Z, Yan J, Li H (2016). Identification and characterization of ovary development-related protein EJO1 (Eri s 2) from the ovary of *Eriocheir sinensis* as a new food allergen. Molecular Nutrition & Food Research.

