# Supplementary figures and images for: Comparison between cultured and wild Pacific white shrimp (Penaeus vannamei) vitellogenesis: next-generation sequencing and relative expression of genes directly and indirectly related to reproduction
Source: PeerJ. 2021 Feb 23;9:e10694. doi: 10.7717/peerj.10694 (PMC7908874; doi:10.7717/peerj.10694)

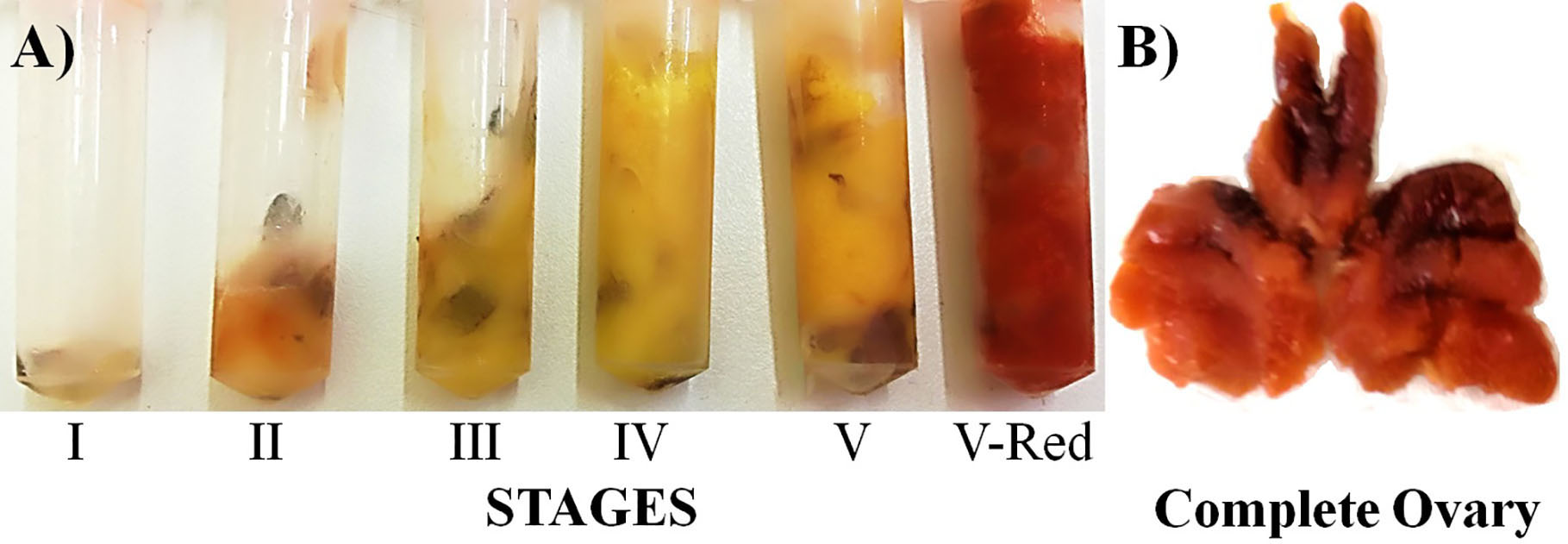

Supplement: Supplemental Information 2 — (A) colorimetric scale. (B) complete gonad from stage V-Red. [file peerj-09-10694-s002.png]
